# Hyperexcitability and Loss of Feedforward Inhibition Contribute to Aberrant Plasticity in the *Fmr1*KO Amygdala

**DOI:** 10.1523/ENEURO.0113-21.2021

**Published:** 2021-05-07

**Authors:** Matthew N. Svalina, E. Mae Guthman, Christian A. Cea-Del Rio, J. Keenan Kushner, Serapio M. Baca, Diego Restrepo, Molly M. Huntsman

**Affiliations:** 1 Neuroscience Graduate Program, University of Colorado Anschutz Medical Campus, Aurora, CO 80045; 2 Department of Pharmaceutical Sciences, Skaggs School of Pharmacy and Pharmaceutical Sciences, University of Colorado Anschutz Medical Campus, Aurora, CO 80045; 3 Medical Scientist Training Program, University of Colorado Anschutz Medical Campus, Aurora, CO 80045; 4 Department of Cell and Developmental Biology, University of Colorado Anschutz Medical Campus, Aurora, CO 80045; 5 Centro de Investigación Biomédica y Aplicada, Escuela de Medicina, Facultad de Ciencias Médicas, Universidad de Santiago de Chile, Santiago 9160000, Chile; 6 Department of Pediatrics, School of Medicine, University of Colorado Anschutz Medical Campus, Aurora, CO 80045

**Keywords:** E/I balance, feed-forward inhibition, fragile X syndrome, lateral amygdala, synaptic plasticity

## Abstract

Fragile X syndrome (FXS) is a neurodevelopmental disorder (NDD) characterized by intellectual disability, autism spectrum disorders (ASDs), and anxiety disorders. The disruption in the function of the *FMR1* gene results in a range of alterations in cellular and synaptic function. Previous studies have identified dynamic alterations in inhibitory neurotransmission in early postnatal development in the amygdala of the mouse model of FXS. However, little is known about how these changes alter microcircuit development and plasticity in the lateral amygdala (LA). Using whole-cell patch clamp electrophysiology, we demonstrate that principal neurons (PNs) in the LA exhibit hyperexcitability with a concomitant increase in the synaptic strength of excitatory synapses in the BLA. Further, reduced feed-forward inhibition appears to enhance synaptic plasticity in the FXS amygdala. These results demonstrate that plasticity is enhanced in the amygdala of the juvenile *Fmr1* knock-out (KO) mouse and that E/I imbalance may underpin anxiety disorders commonly seen in FXS and ASDs.

## Significance Statement

These studies identify significant cellular and synaptic defects in a behaviorally-relevant brain to the pathology of fragile X syndrome (FXS). We find that principal neurons (PNs) in the FXS basolateral amygdala (BLA) exhibit marked hyperexcitability as early as P21. Further, we show that feed-forward inhibition is reduced in the *Fmr1* knock-out (KO) LA. This contributes to enhanced synaptic plasticity in LA of the Fmr1KO mouse.

## Introduction

Fragile X syndrome (FXS) is the most common monogenic form of intellectual disability. FXS is a neurodevelopmental disorder (NDD) broadly characterized by neurologic and psychiatric disorders such as attention deficit hyperactivity disorder, anxiety, social avoidance, increased incidence of seizures and epilepsy, and autism spectrum disorders (ASDs; Hagerman et al., 2009). In humans, FXS is caused by a repeat expansion mutation in the *FMR1* gene that encodes fragile X mental retardation protein (FMRP; Liu et al., 2018). Trinucleotide repeat expansion results in hypermethylation at the *FMR1* locus and subsequent transcriptional silencing of FMRP (Fu et al., 1991). FMRP is an RNA binding protein with a known role in regulating messenger RNA translation during synaptic development (Chen et al., 2003; Darnell et al., 2011). The dysregulation of protein synthesis observed in the pathogenesis of FXS is known to result in significant defects in neuronal development, synaptic and circuit function (Contractor et al., 2015).

In FXS, profound alterations in excitatory and inhibitory neurotransmission have been found across multiple brain regions including the hippocampus, somatosensory cortex, and the basolateral amygdala (BLA; Huber et al., 2002; Olmos-Serrano et al., 2010; Paluszkiewicz et al., 2011; Contractor et al., 2015). Indeed, accumulating evidence directly implicates BLA dysfunction as a key component of many behavioral manifestations in FXS as well as NDDs (Baron-Cohen et al., 2000; Hessl et al., 2004; Bauman and Kemper, 2005; Dalton et al., 2005). Amygdala-based behaviors, including anxiety disorders and social withdrawal, are commonly diagnosed psychiatric disorders in individuals with FXS and ASDs (Tsiouris and Brown, 2004; Turk et al., 2005; Cordeiro et al., 2011). In NDDs such as FXS, patients have increased anxiety and an increased retention of fearful memories (Turk et al., 2005). The adherence to fearful memories dictates the emotional state of the patient (Turk et al., 2005) and likely exacerbates already increased anxiety levels (Meredith et al., 2012). Further, patients with intellectual disabilities can exhibit stress and anxiety from an overactive response to fearful memories, similar to posttraumatic stress and panic disorders (Turk et al., 2005; Roberts et al., 2009).

The amygdala is a grouping of many distinct, heterogeneous nuclei responsible for the integration and processing of information with emotional and social salience (Duvarci and Pare, 2014; Janak and Tye, 2015; Li et al., 2017). Specifically, a large body of work has identified the BLA as the main site of synaptic plasticity underlying the acquisition, expression, and extinction of sensory-threat associations with the BLA also implicated in neuropsychiatric diseases such as anxiety disorders (Duvarci and Pare, 2014; Janak and Tye, 2015). At the cellular level, the BLA is composed of excitatory principal neurons (PNs) and a diverse population of GABAergic inhibitory interneurons (INs; McDonald, 1984; Sah et al., 2003; Duvarci and Pare, 2014). These local PNs undergo input-specific, activity-dependent plastic changes in response to co-occurring threatening and sensory stimuli (Quirk et al., 1995; McKernan and Shinnick-Gallagher, 1997; Schoenbaum et al., 1999; Nabavi et al., 2014; Grewe et al., 2017; Kim and Cho, 2017; Kasugai et al., 2019). Importantly, these co-occurring stimuli depolarize and drive ensembles of BLA PNs to fire action potentials (APs), and this excitation is necessary for learning to occur (Rosenkranz and Grace, 2002; Wolff et al., 2014; Grewe et al., 2017). The local circuit INs play an important role regulating the synaptic plasticity underlying this *in vivo* learning process (Bissière et al., 2003; Wolff et al., 2014). Our group and others have shown this inhibitory control is exerted via a feedforward circuit motif from somatostatin-expressing (Sst) INs which modulates long-term potentiation (LTP) at cortical and thalamic afferent synapses onto BLA PNs (Smith et al., 2000; Bissière et al., 2003; Tully et al., 2007; Unal et al., 2014; Wolff et al., 2014; Bazelot et al., 2015; Ito et al., 2019; Guthman et al., 2020). Thus, feedforward inhibition (FFI)-gated LTP is an underlying circuit mechanism for the acquisition of threat conditioning.

In FXS, profound alterations in the GABAergic system have been previously identified in cortex, hippocampus, brainstem, and BLA (El Idrissi et al., 2005; D’Hulst et al., 2006; Gibson et al., 2008; Olmos-Serrano et al., 2010; Paluszkiewicz et al., 2011; Vislay et al., 2013; Martin et al., 2014). Specifically, prior work from our group has shown reductions in both tonic and phasic inhibitory neurotransmission as well as GABA availability have been observed in the rodent BLA (Olmos-Serrano et al., 2010; Martin et al., 2014), the BLA subnucleus where these fundamental plasticity processes first occur in the canonical BLA circuit (Duvarci and Pare, 2014; Janak and Tye, 2015). However, despite the prevalence of amygdala-based disorders in FXS, the synaptic underpinnings remain unclear as there is limited understanding of the role of diminished inhibition in mediating plasticity in this well-defined circuit responsible for sensory-threat processing. Thus, a better understanding of how heightened anxiety may stem from maladaptive plasticity is essential to identifying new therapeutic avenues.

In the present study, we used whole-cell patch clamp electrophysiology to explore the intrinsic properties of LA PNs, local microcircuit excitation-inhibition (E/I) balance, and synaptic plasticity. We found that excitatory PNs in the *Fmr1* knock-out (KO) LA show marked hyperexcitability compared with wild-type (WT) animals. Consistent with the role of FFI in gating LTP in the LA, we show a correlated loss of FFI and enhanced LTP in the *Fmr1*KO LA. These results demonstrate that altered E/I balance in *Fmr1*KO mice enhances synaptic plasticity in the LA and may underpin behavioral disorders seen in both children with FXS and ASDs.

## Materials and Methods

### Ethical approval

All experiments and procedures were conducted in accordance with protocols approved and reviewed by the Institutional Animal Care and Use Committee (#00039, valid through 3/21/2022) at the University of Colorado Anschutz Medical Campus, in accordance with guidelines from the National Institutes of Health.

### Experimental model and subject details

Slice electrophysiology experiments were conducted on mice aged postnatal day (P)21–P35. Experiments were conducted on both male (*Fmr1^-/y^*) and female (*Fmr1*−/−) mice. The following mouse lines were used in the experiments: C57Bl/6J (The Jackson Laboratory #000664) and FVB.NH (The Jackson Laboratory #001800) and B6.129P2-Fmr1^tm1Cgr^/J (The Jackson Laboratory #003025), and FVB.129P2-Pde6b^+^Tyr^c-ch^Fmr1^tm1Cgr^/J (The Jackson Laboratory #004624). All mice were obtained from The Jackson Laboratory and housed in polypropylene cages with wood shavings with a modified 10/14 h light/dark cycle. Food and water were available *ad libitum*.

### Acute slice preparation for electrophysiology

Mice aged P21–P35 were killed by exposure to a rising concentration of carbon dioxide (CO_2_) at 0.5–1.0 pound per square inch at a 0.5-l/min flow rate until loss of consciousness followed by decapitation. Brains were quickly removed by dissection and glued cerebellar side-down on a vibratome (Leica Biosystems) stage and immersed in an ice-cold and oxygenated cutting solution (95% O_2_/5% CO_2_; 45 mm sucrose, 25 mm glucose, 85 mm NaCl, 2.5 mm KCl, 1.25 mm NaH_2_PO_4_, 25 mm NaHCO_3_, 0.5 mm CaCl_2_, and 7 mm MgCl_2_, osmolality, 290–300 mOsm/kg). We prepared acute coronal slices (300 μm) containing BLA and incubated the slices in oxygenated (95% O_2_/5% CO_2_) artificial CSF (ACSF; 10 mm glucose, 124 mm NaCl, 2.5 mm KCl, 1.25 mm NaH_2_PO_4_, 25 mm NaHCO_3_, 2 mm CaCl_2_, and 2 mm MgCl_2_, osmolality 290–300 mOsm/kg) at 36°C for at least 30 min. All reagents were purchased from Sigma-Aldrich.

#### Electrophysiology

Slices were placed in a submerged slice chamber and perfused with ACSF heated to 32–34°C at a rate of 2 ml/min. Slices were visualized using a moving stage microscope (Scientifica; Olympus) equipped with 4× (0.10 NA) and 40× (0.80 NA) objectives, differential interference contrast (DIC) optics, infrared illumination, LED illumination (CoolLED), a CoolSNAP EZ camera (Photometrics), and Micro-Manager 1.4 (Open Imaging). Whole cell patch clamp recordings were made using borosilicate glass pipettes (2.5–5.0 MW; King Precision Glass) filled with an intracellular recording solution. Data were acquired with a Multiclamp 700B amplifier and were converted to a digital signal with the Digidata 1440 digitizer using pCLAMP 10.6 software (Molecular Devices).

Recordings were obtained from visually identified excitatory PNs in the LA. PNs were targeted based on their large, pyramidal-like soma and strict biophysical criteria based on Sah et al., 2003) and our previous studies (Olmos-Serrano et al., 2010; Vislay et al., 2013; Martin et al., 2014). For voltage clamp experiments, a cesium methanesulfonate (CsMe) based intracellular solution was used (120 mm CsMe, 10 mm HEPES, 0.5 mm EGTA, 8 mm NaCl, 10 mm Na-phosphocreatine, 1 mm QX-314, 4 mm MgATP, and 0.4 mm Na_2_GTP, pH to 7.3 with CsOH; osmolality adjusted to ∼290 mOsm/kg). For all current clamp and plasticity experiments, a potassium gluconate based intracellular solution was used (135 mm potassium gluconate, 10 mm HEPES, 20 mm KCl, 0.1 mm EGTA, 2 mm MgATP, and 0.3 mm Na_2_GTP, pH to 7.3 with KOH; osmolality adjusted to ∼295 mOsm/kg). Access resistance was monitored throughout the experiments and data were discarded if access resistance exceeded 25 MΩ or varied by >20%. No junction potential compensation was performed. Series resistance was not compensated in either voltage or current clamp. In current clamp, compensation for voltage variations was achieved using a bridge balance circuit. In voltage clamp, during recording of small synaptic (spontaneous and evoked) events, series resistance was monitored throughout the experiment. Data were sampled at 10 kHz and lowpass filtered at 4 kHz. Offline, current data were filtered using either a third order Savistky–Golay filter with a ±0.5-ms window or a 2-kHz lowpass butterworth filter. Mean traces were created by first aligning all events by their point of maximal rise (postsynaptic currents) and then obtaining the mean of all events.

#### Electrophysiology experimental design

##### Ramped current injections

Immediately after achieving whole-cell configuration, LA neurons were recorded at rest in current clamp mode (I_hold_ = 0 pA). Following a three second baseline period, the holding current was linearly ramped from 0 to 400 pA over 2 s. A total of 25 sweeps of data were collected for each neuron, and the data were used to determine the resting membrane potential (V_rest_), AP threshold, and rheobase current of LA PNs.

##### Square current injections

Following ramped current injections, we recorded the responses of LA neurons to a series of square hyperpolarizing and depolarizing current injections. Before initiation of the series of current injections, V_m_ of the LA neurons was adjusted to approximately −60 mV. Each cell was subjected to two series of 600-ms square current injections: −100 to +100 pA at 10-pA intervals and −200 to +400 pA at 25-pA intervals. The data collected in these experiments were used to determine active and passive membrane properties of the neurons.

##### Spontaneous (s)EPSCs/sIPSCs

sEPSCs (V_hold_ = −70 mV) and sIPSCs (V_hold_ = 0 mV) in LA PNs were recorded for 80 s each.

##### Input-output curves

Thalamic afferents from the internal capsule were stimulated using a bipolar stimulating electrode (FHC). We recorded evoked EPSCs (V_hold_ = −70 mV) and IPSCs (V_hold_ = 0 mV) from LA PNs in response to internal capsule stimulation. Experiments were conducted over a range of stimulation intensities (0–100 μA with a 10-μA interval).

##### Paired-pulse EPSC experiments

Thalamic afferents from the internal capsule were stimulated twice at 10 and 50 Hz (100- and 20-ms interstimulus intervals) at a 100-μA stimulus intensity. Evoked EPSCs (V_hold_ = −70 mV) from LA PNs in response to this stimulation.

##### Synaptic plasticity

For LTP experiments, we recorded AMPA mediated-EPSCs elicited by electrical stimulation of the internal capsule (stimulation frequency = 0.066 Hz) in LA PNs from WT and *Fmr1*KO mice at P21–P35 (voltage-clamp configuration, V_hold_ = –80 mV) in the presence or absence of the GABA_A_ receptor antagonist, gabazine (SR-95531, 10 μm in DMSO, Tocris Biosciences). Following a 5-min baseline recording, high-frequency electrical stimulation (HFS; two trains of 100 pulses delivered at 100 Hz, 20 s apart) were delivered to the internal capsule. EPSCs were measured for 20–45 min after HFS in the same way as baseline recordings. Synaptic strength was quantified as the integrated charge of each evoked EPSC. Change in synaptic strength was determined by normalizing the integrated charge of each EPSC recorded both before and after HFS to the average integrated charge of all baseline recordings (average normalized integrated charge of baseline = 100%). Successful LTP induction was defined as a significant increase in normalized integrated charge during the last 5 min (minutes 16–20) after HFS compared with baseline (minutes −5 to −1).

#### Definitions of electrophysiological parameters

##### V_rest_

V_rest_ was defined as the mean V_m_ (I_hold_ = 0 pA) during a 500-ms baseline across all sweeps in the ramped injection experiments.

##### AP threshold

AP threshold was defined as the voltage at which dV/dt exceeded 20 V/s. AP threshold was calculated at the first AP of each sweep in the ramped injection experiments.

##### Rheobase current

Rheobase current was defined as the mean current injected at AP threshold for the first AP across all sweeps in the ramped injection experiments.

##### Membrane resistance (R_m_)

R_m_ was defined as the slope of the best fit line of the I-V plot using the −100 to +100 pA (10-pA steps) series of current injections. Mean voltage response to each current injection step was defined as the difference between baseline mean membrane voltage (100 ms before current injection) and the mean membrane voltage during the 100-ms period from 50 ms after the start of the injection to 150 ms after the start of the current injection. This 100-ms window was chosen to allow for measurement of the change in V_m_ after the membrane had charged and before any potential HCN channel activation. The I-V plot was constructed using all current steps below rheobase.

##### Maximum firing rate

Maximum firing rate was defined as the inverse of the interspike interval (ISI) during the first 200 ms of the most depolarizing current injection step before attenuation of AP firing was observed. Maximum firing rate was calculated using the −200 to +400 pA (25-pA steps) series of current injections.

##### AP amplitude

Amplitude of the AP was defined as the voltage difference between the peak of the AP and its threshold potential (set at dV/dt = 20 V/s). AP amplitude was calculated at the rheobase sweep of the −200 to +400 pA (25-pA steps) series of current injections.

##### AP halfwidth

AP halfwidth was defined as the time between the half-amplitude point on the upslope of the AP waveform to the half-amplitude point on the downslope of the AP waveform. AP halfwidth was calculated at the rheobase sweep of the 200 to +400 pA (25-pA steps) series of current injections.

##### After-hyperpolarization potential (AHP) magnitude

AHP magnitude was defined as the difference between the most hyperpolarized membrane voltage of the AHP (occurring within 100 ms after AP threshold) and AP threshold. AHP magnitude and latency data were calculated at the rheobase sweep of the −200 to +400 pA (25-pA steps) series of current injections. ΔAHP data were calculated at the rheobase +50-pA sweep of the −200 to +400 pA (25-pA steps) series of current injections.

##### AHP latency

AHP latency was defined as the time from AP threshold and the peak of the AHP.

##### ΔAHP

ΔAHP was defined as the difference between the first and last AHP (ΔAHP = AHP_last_ – AHP_first_).

##### AP phase plot

The AP phase plot was obtained by plotting the rate of change of the mean AP for each cell from the rheobase sweep of the −200 to +400 pA (25-pA steps) series of current injections as a function of the corresponding membrane voltage.

##### Latency to first AP

AP latency was defined as the time from the initiation of the current injection to the peak of the first AP. AP latency was calculated at the rheobase sweep of the −200 to +400 pA (25-pA steps) series of current injections.

##### Firing rate adaptation ratio

Firing rate adaptation was defined as the ratio of the first and the average of the last two ISIs, such that firing rate adaptation = ISI_first_/meanISI_last two ISI_. Firing rate adaptation was calculated at the rheobase +50-pA sweep of the −200 to +400 pA (25-pA steps) series of current injections.

##### AP broadening

AP broadening was defined as the ratio of the AP halfwidths of the first two APs (broadening = halfwidth_second_/halfwidth_first_). AP broadening was calculated at the rheobase +50-pA sweep of the −200 to +400 pA (25-pA steps) series of current injections.

##### AP amplitude adaptation

AP amplitude adaptation was defined as the ratio of the AP amplitude of the average of the last three APs and the first AP, such that AP amplitude adaptation = meanamplitude_last 3 APs_/amplitude_first AP_. AP amplitude adaptation was calculated at the rheobase +50-pA sweep of the −200 to +400 pA (25-pA steps) series of current injections.

##### Membrane decay τ

Membrane decay τ was determined by using a single exponential fit, f(t) = A$e^{-t/\tau }$ to fit the change in V_m_ induced by a −100-pA sweep in the −100 to +100 pA (25-pA steps) series of current injections.

##### Hyperpolarization-induced sag

Hyperpolarization-induced sag was calculated using the equation, $\frac{Vmin-Vss}{Vmin-Vbl}\times$100%, where V_min_ was defined as the most hyperpolarized membrane voltage during the current injection, V_ss_ was defined as the mean steady-state membrane voltage (last 200 ms of the current injection), and V_bl_ was defined as the mean baseline membrane voltage (100 ms before current injection). Hyperpolarization-induced sag was measured from the −200-pA current injection.

##### Rebound spikes

Rebound spikes were defined as the number of APs in the 500 ms following the −200-pA current injection.

##### sEPSC/IPSC detection and amplitude

sEPSC/IPSCs were detected by a combined template and threshold method. Briefly, a template was made by subsampling 10% of local peaks exceeding at least 6× or 7× (sEPSC or sIPSC, respectively) the median absolute deviation of a rolling baseline current (50 ms before the peak). The template current was then truncated from its 20% rise point through the end of the decay time constant for the template current. Next, all local peaks exceeding 6× or 7× the median absolute deviation of a rolling baseline current (50 ms before the peak) were collected. The template was then scaled to each individual putative sEPSC or sIPSC peak and each peak was assigned a normalized charge integral relative to the template. Finally, a normalized charge integral cutoff was chosen to exclude obvious noise/non-physiological events below a certain normalized charge integral. sEPSC amplitude was defined as the difference between the peak amplitude of each detected current and its corresponding baseline current. sEPSC/IPSC amplitude for each cell was defined as the median peak amplitude for that cell. sEPSC/IPSC frequency was defined as the inverse of the interevent intervals of the events. The frequency measure for each neuron was defined as the median of the sEPSC/IPSC frequencies for that cell.

##### sEPSC/IPSC 20–80% risetime

A total of 20–80% risetime was defined as the time it took an sEPSC or sIPSC to reach 80% of its peak amplitude from 20% of its peak amplitude; 20–80% risetime was calculated from the mean sEPSC/sIPSC of a given LA PN.

##### EPSC/IPSC τ_Decay_

EPSC τ_Decay_ was determined using a single exponential fit, f(t) = A$e^{-t/\tau }$. IPSC τ_Decay_ was defined as the weighted time-constant of IPSC decay. Briefly, a double exponential fit, f(t) = A_1_$e^{-t/\tau 1}$+ A_2_$e^{-t/\tau 2}$, was used to obtain the parameters to determine the weighted time-constant where τ_Weighted_ = (τ_1_A_1_ + τ_2_A_2_)/(A_1_ + A_2_). For spontaneous events, the mean cellular EPSC or IPSC was used to determine the decay kinetics. For evoked events, the mean cellular EPSC or IPSC in response to 100-μA stimulation was used to determine the decay kinetics.

##### EPSC/IPSC detection and amplitude, input-output curves

To determine the evoked EPSC and IPSC amplitudes across varying stimulus intensities, we first determined the peak time relative to the 100-μA stimulation. Then, we defined EPSC or IPSC amplitude as the maximum positive or negative deflection, respectively, from the mean current response within a window of 6 SDs of the peak time jitter.

##### Stimulation for half-maximum EPSC/IPSC amplitude

To get the half-maximum stimulation intensity and the slope of the input-output curve, we used the least squares method to fit a line to the EPSC/IPSC output relative to stimulation input. We only used input values that elicited non-zero EPSC/IPSC amplitudes to determine the best fit line. We then used this best fit line to find the stimulation intensity that was associated with 50% of the maximum EPSC/IPSC amplitude for the PN.

##### Input-output curve slope

We defined input-output slope as the slope of the line created with a least squares fit of the input-output curve.

##### Paired-pulse ratio

To determine paired pulse ratio, we first determined the peak amplitude of the maximum negative deflection from the mean current trace during the poststimulus period (20 or 100 ms poststimulation) for each of the paired stimulations. We defined the paired-pulse ratio such that paired-pulse ratio = amplitude_EPSC,second_/amplitude_EPSC,first_.

### Statistics

#### Statistical analyses

All data analysis was performed using custom written MATLAB code and GraphPad Prism. Normality of the data were assessed using the Anderson–Darling test. For a test between two groups normal data, an unpaired *t* test was used. For tests between two groups of non-normal data, a Mann–Whitney *U* (MWU) test was used. For examination of paired pulse experiment results across genotype, a two-way repeated measures ANOVA was used. Genotype was used as the between-subjects model and interstimulus interval was used as the within-subjects model. All statistical tests were two-tailed. Unless otherwise stated, experimental numbers are reported as *n *=* *x, y, where x is the number of neurons and y is the number of mice.

#### Data display

Data visualizations were created in MATLAB, GraphPad Prism and Adobe Illustrator. Normal data are presented as the mean ± SD. Non-normal data are presented as the median with error bars extending along the interquartile range.

### Data and software availability statement

Data and code are available on request, and code will be made available on GitHub at https://github.com/emguthman.

### Reagent and resource sharing

Further information and requests for resources and reagents should be directed to and will be fulfilled by corresponding author.

## Results

### LA PNs in *Fmr1*KO mice exhibit marked hyperexcitability

**Figure 1.**
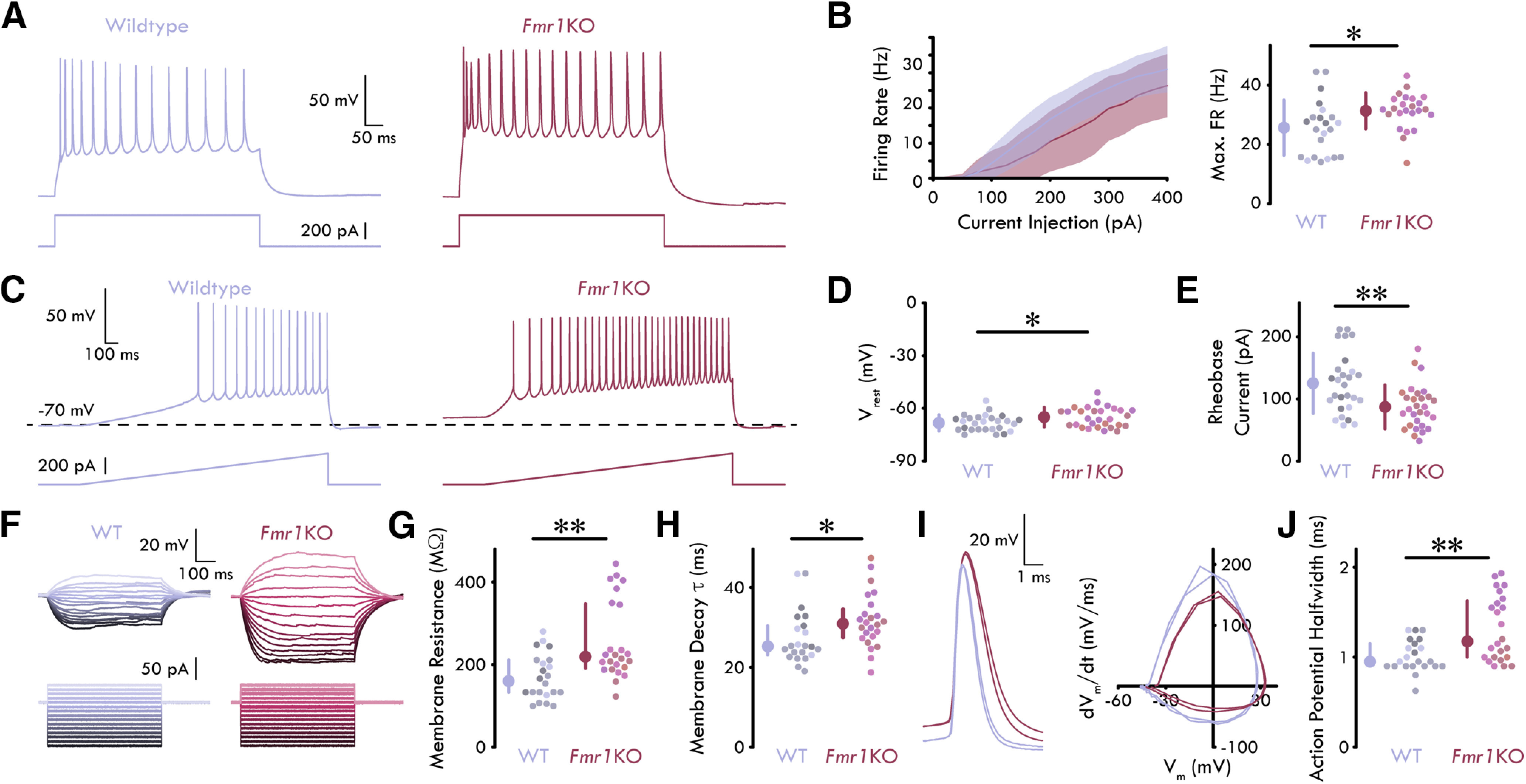
Hyperexcitability of PNs in *Fmr1*KO LA. ***A***, Representative traces of maximum firing rate response to rectangular current injections in WT and *Fmr1*KO LA PNs. ***B***, left, Mean firing rate of LA PNs. Shading shows SD. Right, Maximum firing rate of LA PNs is greater in *Fmr1*KO compared with WT LA [unpaired *t* test: *p *=* *0.0175; *n*_WT_ = 22 neurons, 3 mice, 2 males (M), 1 female (F); *n_Fmr1_*_KO_ = 24, 4, 3 M, 1 F]. ***C***, Representative traces of voltage response to a ramped current injection in WT and *Fmr1*KO LA PNs. ***D***, *Fmr1*KO LA PNs have a more depolarized V_rest_ compared with WT LA PNs (unpaired *t* test: *p* = 0.0189; *n*_WT_ = 27, 4, 3 M, 1 F; *n_Fmr1_*_KO_ = 29, 4, 3 M, 1 F). ***E***, *Fmr1*KO LA PNs have a lower rheobase current from rest compared with WT LA PNs (unpaired *t* test: *p *=* *0.00135; *n*_WT_ = 27, 4, 3 M, 1 F; *n_Fmr1_*_KO_ = 29, 4, 3 M, 1 F). ***F***, Representative traces of voltage responses to intermediate current injection traces used to determine R_m_ and decay τ (−100 to +100 pA; Δ10 pA). ***G***, R_m_ is increased in *Fmr1*KO LA PNs compared with WT LA PNs (MWU test: *p *=* *0.0016; *n*_WT_ = 22, 3, 2 M, 1 F; *n_Fmr1_*_KO_ = 24, 4, 3 M, 1 F). ***H***, Membrane decay τ is increased in *Fmr1*KO LA PNs compared with WT LA PNs (MWU test: *p *=* *0.00160; *n*_WT_ = 22, 3, 2 M, 1 F; *n_Fmr1_*_KO_ = 24, 4, 3 M, 1 F). ***I***, Left, Representative AP traces from a WT and *Fmr1*KO LA PN at rheobase current injection. Right, Phase plot for the same APs. ***J***, *Fmr1*KO LA PNs have broader AP halfwidths compared with WT LA PNs (MWU test: *p *=* *0.00336; *n*_WT_ = 22, 3, 2 M, 1 F; *n_Fmr1_*_KO_ = 24, 4, 3 M, 1 F). Summary statistics in ***B***, ***D***, ***E*** presented as mean ± SD. Summary statistics in ***H***, ***J*** presented as median with IQR; *p < 0.05, **p < 0.01. Individual neurons plotted and represented by different colors on a per animal basis.

To examine potential differences in neuronal excitability, we prepared acute coronal brain slices containing the BLA. We performed whole cell patch-clamp recordings of LA PNs and compared their intrinsic biophysical properties across WT and *Fmr1*KO juvenile mice (P21–P35). In these experiments, we measured 18 membrane properties (Fig. 1; Table 1) by examining voltage responses to both a ramped and rectangle current injections (see Materials and Methods).

**Table 1:** Differences in active and passive membrane properties among LA PNs in WT and Fmr1KO mice.

	WT PNs (*^a^n* = 27; *^b^n* = 22)		*Fmr1*KO PNs (*^a^n* = 29; *^b^n* = 24)		Statistical comparisons
	Mean/median	Variance	Mean/median	Variance	
*^a^*Resting membrane voltage (mV)	−68.33	±4.63	−64.99	±5.60	*p *=* *0.0189, unpaired *t* test
*^a^*Rheobase current (pA)	125.33	±48.48	87.21	±35.30	*p *=* *0.00135, unpaired *t* test
*^a^*AP threshold (mV)	−35.75	±3.59	−35.50	±4.90	*p *=* *0.827, unpaired *t* test
*^b^*R_m_ (MW)	159.89	132.75/211.01	218.94	190.68/347.18	*p *=* *0.00160, MWU test
*^b^*τ_Membrane_ (ms)	25.24	23.08/30.38	30.92	27.50/34.49	*p *=* *0.00160, MWU test
*^b^*Maximum firing rate (Hz)	25.66	±9.34	31.37	±6.14	*p *=* *0.0175, unpaired *t* test
*^b^*AP halfwidth (ms)	0.95	0.90/1.15	1.18	1.00/1.63	*p *=* *0.00336, MWU test
*^b^*AP threshold (mV)	−35.75	±0.69	−35.50	±0.91	*p *=* *0.827, unpaired *t* test
*^b^*Latency to first AP (ms)	125.60	111.30/167.40	137.15	110.40/175.30	*p *=* *0.531, MWU test
*^b^*Firing rate adaptation	0.54	±0.14	0.54	±0.12	*p *=* *0.928, unpaired *t* test
*^b^*AP broadening	1.12	1.08/1.25	1.20	1.11/1.34	*p *=* *0.158, MWU test
*^b^*AP amplitude (mV)	69.56	±8.40	66.17	±13.15	*p *=* *0.309, unpaired *t* test
*^b^*Amplitude adaptation	0.96	0.91/0.98	0.96	0.93/0.99	*p *=* *0.767, MWU test
*^b^*AHP magnitude (mV)	17.57	±2.98	18.65	±2.91	*p *=* *0.219, unpaired *t* test
*^b^*ΔAHP (mV)	−2.76	−2.44/−3.99	−3.61	−2.38/−3.61	*p *=* *0.517, MWU test
*^b^*AHP latency (ms)	46.83	±15.31	50.97	±11.97	*p *=* *0.311, unpaired *t* test
*^b^*Hyperpolarization-induced sag (%)	6.24	5.19/7.96	8.32	4.31/9.59	*p *=* *0.621, MWU test
*^b^*Rebound APs	0.00	0.00/0.00	0.00	0.00/0.00	*p *=* *1.00, MWU test

Normal data are presented as mean ± SD with differences tested using an unpaired *t* test. Non-normal data are presented as median and IQR with differences tested using a MWU test.

We observed significant differences in both active and passive membrane properties of LA PNs in *Fmr1*KO compared with WT animals. Specifically, depolarizing current injections drove increased AP firing rates in LA PNs of *Fmr1*KO compared with WT animals [unpaired *t* test: *p *=* *0.0175, mean difference (MD): 5.71 Hz, confidence interval (CI): [−10.4, −1.05]; *n*_WT_ = 22 neurons, three mice; *n_Fmr1_*_KO_ = 24, 4; Fig. 1*A*,*B*]. Additionally, PNs in the LA of *Fmr1*KO mice exhibited a higher V_rest_ and a lower rheobase current compared with PNs in WT mice (V_rest_, unpaired *t* test: *p *=* *0.0189, MD: −3.34 mV, CI: [−6.10, −0.57]; rheobase current: unpaired *t* test: *p *=* *0.00135, MD: 38.12 pA, CI: [15.5, 60.7], *n*_WT_ = 22, 3; *n_Fmr1_*_KO_ = 24, 4; Fig. 1*C–E*). Further, LA PNs in *Fmr1*KO mice showed increased R_m_, increased membrane decay τ, and AP halfwidth (R_m_, MWU test: *p *=* *0.00160, MD: 5.68 ms, z: −2.41, rank-sum: 407; decay τ, MWU test: *p *=* *0.0016; halfwidth, MWU test: *p *=* *0.00336, MD: 0.28 ms, z: −2.93, rank-sum: 384; *n*_WT_ = 22, 3; *n_Fmr1_*_KO_ = 24, 4; Fig. 1*F–J*). No other membrane property comparisons reached statistical significance (Table 1). Overall, these data reveal increased intrinsic membrane excitability in the LA PNs of *Fmr1*KO compared with WT mice.

### Alterations in spontaneous excitation and inhibition in *Fmr1*KO LA

**Figure 2.**
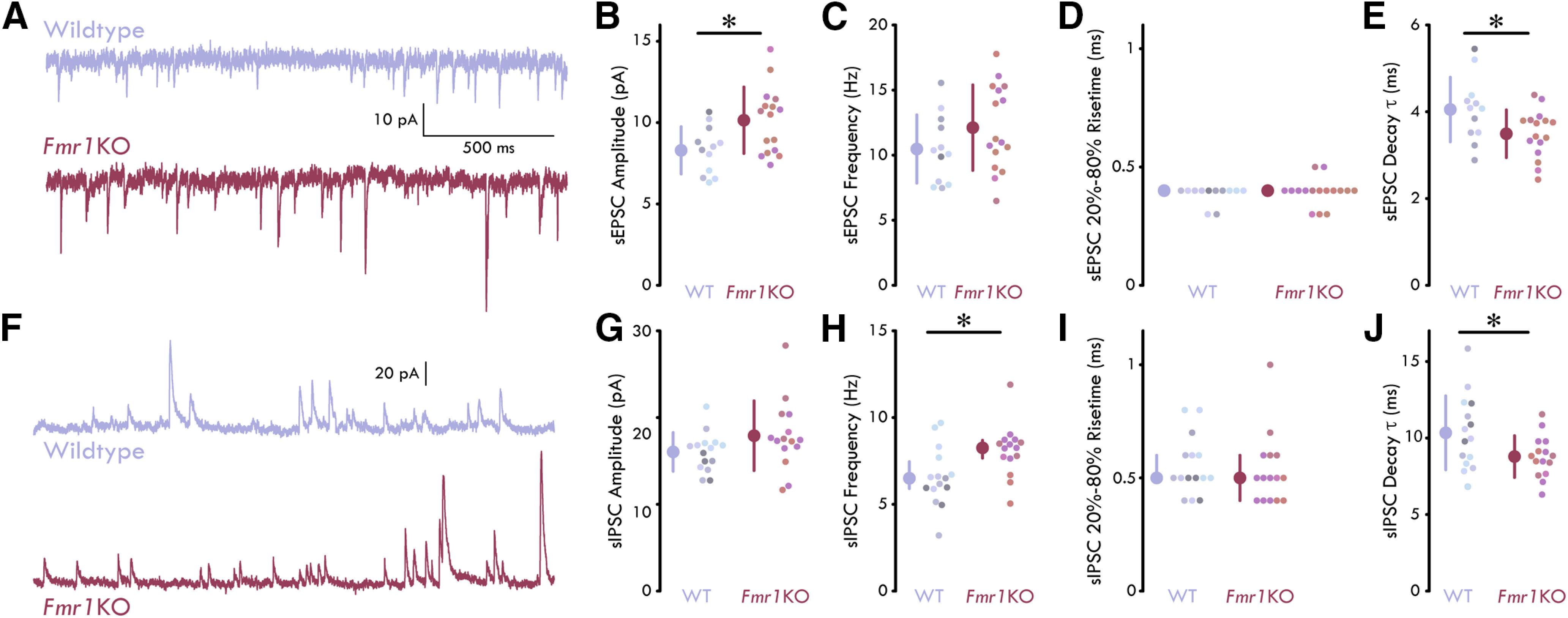
Enhanced sEPSC and sIPSC in *Fmr1*KO LA. ***A***, Representative current traces from LA PNs held at −70 mV. ***B*,** sEPSC amplitude is increased in LA PNs of *Fmr1*KO mice [unpaired *t* test: *p *=* *0.0129; *n*_WT_ = 12 neurons, 7 mice, 7 males (M); *n_Fmr1_*_KO_ = 16, 5, 5 M]. ***C***, No significant difference in sEPSC frequency in LA PNs between WT and *Fmr1*KO mice (unpaired *t* test: *p *=* *0.166; *n*_WT_ = 12, 7, 7 M; *n_Fmr1_*_KO_ = 16, 5, 5 M). ***D***, No significant difference in sEPSC risetime in LA PNs between WT and *Fmr1*KO mice (MWU test: *p *=* *0.646; *n*_WT_ = 12, 7, 7 M; *n_Fmr1_*_KO_ = 16, 5, 5 M). ***E***, sEPSC decay τ is reduced in LA PNs of *Fmr1*KO mice (unpaired *t* test: *p *=* *0.0305; *n*_WT_ = 12, 7, 7 M; *n_Fmr1_*_KO_ = 16, 5, 5 M). ***F***, Representative current traces from LA PNs held at 0 mV. ***G***, No significant difference in sIPSC amplitude in LA PNs between WT and *Fmr1*KO mice (unpaired *t* test: *p *=* *0.127; *n*_WT_ = 15, 7, 7 M; *n_Fmr1_*_KO_ = 15, 4, 4 M). ***H***, sIPSC frequency is increased in LA PNs of *Fmr1*KO mice (MWU test: *p *=* *0.0161; *n*_WT_ = 15, 7, 7 M; *n_Fmr1_*_KO_ = 15, 4, 4 M). ***I***, No significant difference in sIPSC risetime in LA PNs between WT and *Fmr1*KO mice (MWU test: *p *=* *0.504; *n*_WT_ = 15, 7, 7 M; *n_Fmr1_*_KO_ = 15, 4, 4 M). ***J***, sIPSC decay τ is reduced in LA PNs of *Fmr1*KO mice (unpaired *t* test: *p *=* *0.0390; *n*_WT_ = 15, 7, 7 M; *n_Fmr1_*_KO_ = 15, 4, 4 M). Summary statistics in ***C***, ***E***, ***G***, ***J*** presented as mean ± SD. Summary statistics in ***D***, ***H***, ***I*** presented as median with IQR; *p < 0.05. Individual neurons plotted and represented by different colors on a per animal basis.

Previous studies from our group identified defects in BLA inhibitory neurotransmission such that the frequency and amplitude of both phasic and tonic IPSCs are reduced during the P21–P35 development time point (Olmos-Serrano et al., 2010; Vislay et al., 2013; Martin et al., 2014). One possible explanation for this reduction could be that it is a homeostatic response to a concomitant change in sEPSCs. However, our previous studies on synaptic transmission were done in the presence of NMDA and AMPA receptor antagonists (D-APV and DNQX, respectively) to induce a complete excitatory blockade and isolate sIPSCs (Olmos-Serrano et al., 2010; Vislay et al., 2013). To study how loss of *Fmr1* contributes to both spontaneous glutamatergic and GABAergic synaptic transmission in intact LA, we performed patch-clamp recordings in local PNs of *Fmr1*KO and WT animals using a CsMe-based intracellular solution. This solution allows us to voltage clamp the PNs at −70 mV to isolate sEPSCs and 0 mV to isolate sIPSCs (Fig. 2*A*,*F*).

We found that sEPSCs from PNs in *Fmr1*KO LA showed an increase in sEPSC amplitude relative to WT controls (unpaired *t* test, *p *=* *0.0129 MD: 1.35 pA, CI: [0.425, 3.28], *n*_WT_ = 12 neurons, 7 mice; *n_Fmr1_*_KO_ = 16, 5; Fig. 2*B*). We found no differences in the frequency or 20%−80% risetime (frequency: unpaired *t* test, *p *=* *0.166; risetime: MWU test, *p *=* *0.646; *n*_WT_ = 12, 7; *n_Fmr1_*_KO_ = 16, 5; Fig. 2*C*,*D*). Additionally, sEPSCs from PNs in *Fmr1*KO showed a significantly decreased decay τ (unpaired *t* test, *p *=* *0.031, MD: 0.49 ms, CI: [0.0570, 1.06]; *n*_WT_ = 12, 7, *n_Fmr1_*_KO_ = 16, 5; Fig. 2*E*). When we examined sIPSCs, we found no change in sIPSC amplitude; however, we found an increase in the frequency of sIPSCs onto local PNs (amplitude: unpaired *t* test, *p *=* *0.127; frequency: MWU test, *p *=* *0.0161, z: −2.41, rank-sum: 174; *n*_WT_ = 15, 7 *n_Fmr1_*_KO_ = 15, 4; Fig. 2*G*,*H*). As with sEPSCs, we found no significant difference in the 20%−80% risetime of PNs in *Fmr1*KO and WT LA (MWU test, *p *=* *0.504, *n*_WT_ = 15, 7; *n_Fmr1_*_KO_ = 15, 4; Fig. 2*I*). However, LA PNs in *Fmr1*KO mice exhibited a decrease in sIPSC decay (unpaired *t* test, *p *=* *0.0390, MD: 1.17 ms, CI: [0.0842, 3.02], *n*_WT_ = 15, 7, *n_Fmr1_*_KO_ = 15, 4; Fig. 2*J*). Taken together, these data identify differences in presynaptic and postsynaptic modulation of excitatory and inhibitory neurotransmission in the LA of *Fmr1*KO mice.

### FFI is reduced in *Fmr1*KO LA

Disrupted E/I balance of neuronal networks is a hallmark of NDDs (Nelson and Valakh, 2015). In FXS, this manifests as an increased prevalence of anxiety, epilepsy, and attention deficit and hyperactivity (Musumeci et al., 1999; Rogers et al., 2001; Clifford et al., 2007). To further test the hypothesis that loss of *Fmr1* leads to a disruption in local circuit E/I balance, we measured the amplitudes of evoked EPSCs and IPSCs in LA PNs following stimulation of the internal capsule. The internal capsule carries thalamic afferents to the LA (LeDoux et al., 1991). We focused on this afferent synapse as it is a major site of the input-specific LTP that underlies the acquisition of classical Pavlovian threat conditioning *in vivo* (McKernan and Shinnick-Gallagher, 1997; Namburi et al., 2015).

**Figure 3.**
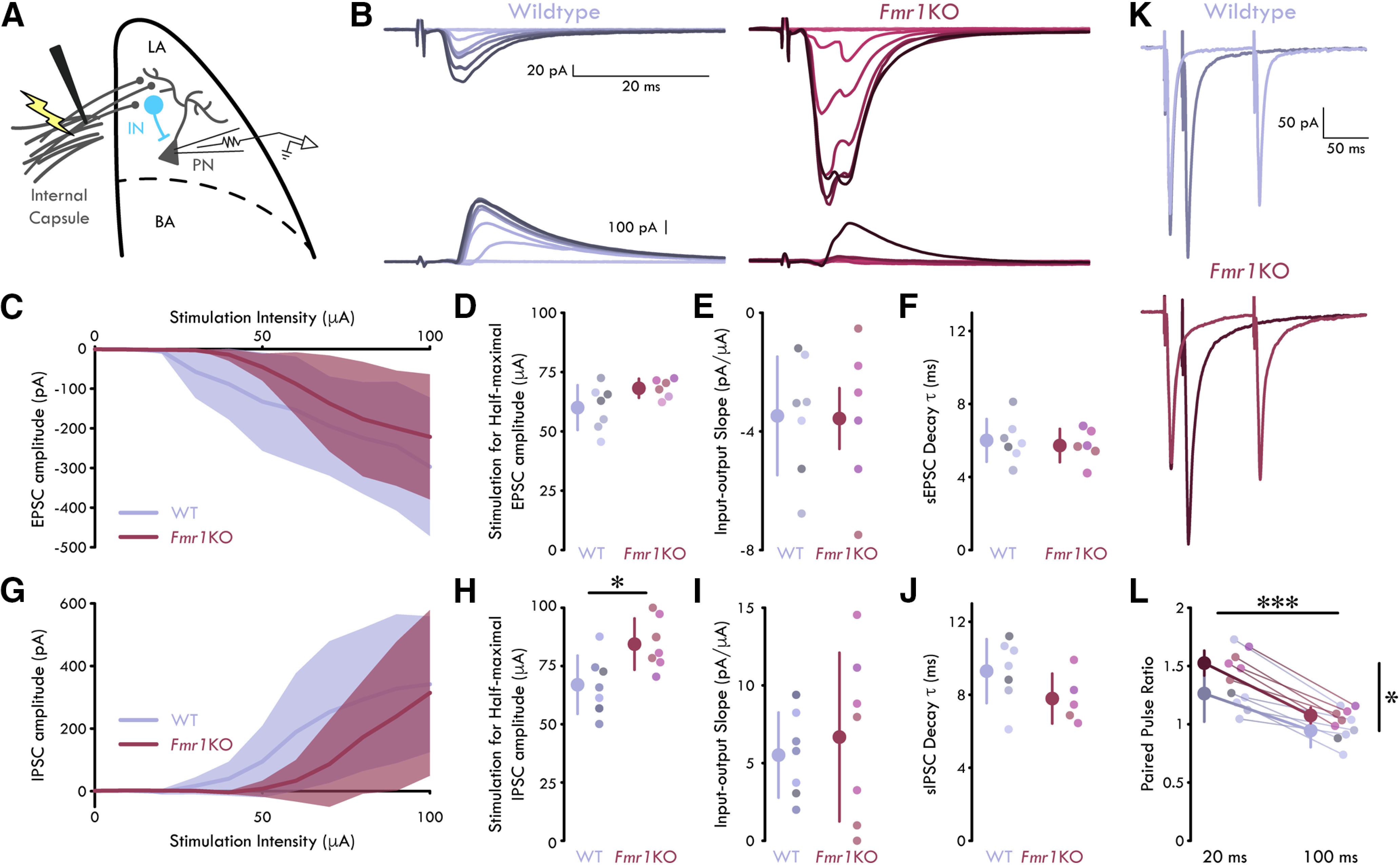
Reduced FFI in *Fmr1*KO LA. ***A***, Experimental schematic. ***B***, Representative mean traces of EPSCs (top) and IPSCs (bottom) in LA PNs following internal capsule stimulation. Color scales with stimulation intensity (light to dark: 0–100 μA). ***C***, Mean evoked EPSC amplitude as a function of stimulation intensity. Shading shows SD. ***D***, Stimulation for half-maximal EPSC amplitude is not significantly different for LA PNs from WT and *Fmr1*KO mice [unpaired *t* test: *p *=* *0.0754; *n*_WT_ = 7 neurons, 5 mice, 5 males (M); *n_Fmr1_*_KO_ = 6, 3, 3 M]. ***E***, Evoked EPSC input-output slope is not significantly different for LA PNs from WT and *Fmr1*KO mice (unpaired *t* test: *p *=* *0.944; *n*_WT_ = 7 neurons, 5 mice, 5 M; *n_Fmr1_*_KO_ = 6, 3, 3 M). ***F***, Evoked EPSC decay τ is not significantly different for LA PNs from WT and *Fmr1*KO mice (unpaired *t* test: *p *=* *0.641; *n*_WT_ = 7, 5, 5 M; *n_Fmr1_*_KO_ = 6, 3, 3 M). ***G***, Mean evoked IPSC amplitude as a function of stimulation intensity. Shading shows SD. ***H***, Stimulation for half-maximal IPSC amplitude is increased for LA PNs from *Fmr1*KO mice (unpaired *t* test: *p *=* *0.0168; *n*_WT_ = 7, 4, 4 M; *n_Fmr1_*_KO_ = 7, 3, 3 M). ***I***, Evoked IPSC input-output slope is not significantly different for LA PNs from WT and *Fmr1*KO mice (unpaired *t* test: *p *=* *0.623; *n*_WT_ = 7, 4, 4 M; *n_Fmr1_*_KO_ = 7, 3, 3 M). ***J***, Evoked IPSC decay τ is not significantly different for LA PNs from WT and *Fmr1*KO mice (unpaired *t* test: *p *=* *0.141; *n*_WT_ = 7, 5, 5 M; *n_Fmr1_*_KO_ = 5, 3, 3 M). ***K***, Representative mean traces of EPSCs in paired-pulse experiments. Darker colors show 20-ms interstimulus interval, and lighter colors show 100-ms interstimulus interval. ***L***, Paired-pulse ratio is increased in PNs from *Fmr1*KO mice and for shorter interstimulus interval durations (two-way repeated measures ANOVA, main effects of genotype and interstimulus interval: *p*_Genotype_ = 0.0465, *p*_interstimulus interval_ = 1.56 × 10^−5^; *n*_WT_ = 6, 4, 4 M; *n_Fmr1_*_KO_ = 5, 3, 3 M). All summary statistics as mean ± SD; *p < 0.05, ***p < 0.001. Individual neurons plotted and represented by different colors on a per animal basis.

Similar to the spontaneous synaptic event experiments above, we isolated evoked EPSC and IPSCs by voltage clamping LA PNs at −70 and 0 mV, respectively. To determine how loss of *Fmr1* affects feedforward excitatory and inhibitory drive onto LA PNs, we recorded evoked EPSCs and IPSCs over a stimulus intensity range of 0–100 μA (Fig. 3*A*,*B*). We found that loss of *Fmr1* had no effect on feedforward excitatory drive to LA PNs as measured by either the stimulation intensity for the half-maximal EPSC amplitude or the slope of the input-output function (halfmax stimulation intensity: unpaired *t* test, *p *=* *0.0754; input-output slope: unpaired *t* test, *p *=* *0.944; decay τ: unpaired *t* test, *p *=* *0.641; *n*_WT_ = 7 neurons, 5 mice; *n_Fmr1_*_KO_ = 6, 3; Fig. 3*A–F*). However, we found that feedforward inhibitory drive was reduced in LA PNs of *Fmr1*KO mice. Specifically, we found that loss of *Fmr1* led to an increase in the stimulation intensity for half-maximal IPSC amplitude in LA PNs (unpaired *t* test, *p *=* *0.0168, MD: 1.48 μA, CI: [−31.1, −3.74]; *n*_WT_ = 7, 4; *n_Fmr1_*_KO_ = 7, 3; Fig. 3*H*). There was no effect of loss of *Fmr1* on the slope of the input-output function or evoked IPSC decay (unpaired *t* test, *p *=* *0.623, *n*_WT_ = 7, 4; *n_Fmr1_*_KO_ = 7, 3; decay τ: unpaired *t* test, *p *=* *0.142, *n*_WT_ = 7, 5; *n_Fmr1_*_KO_ = 5, 3; Fig. 3*I*,*J*). These data indicate that a greater amount of activation of the thalamic afferents to LA is needed to drive similar FFI onto PNs in *Fmr1*KO compared with WT mice. However, the lack of change in input-output slope indicates that once afferent activity is sufficient to elicit IPSCs in the postsynaptic PNs, the IPSC amplitudes increase as a similar function of afferent activity. This finding is in accordance with prior work showing increased feedforward E/I balance in cortical microcircuits *Fmr1*KO mice at the same developmental time point (Antoine et al., 2019). For example, we also observed a modest decrease in evoked excitation. While not statistically significant, this scaling may be biologically significant as computational modeling suggests that a range of mean predicted changes in overall PSP peak serve to maintain stable E/I balance.

Finally, we compared the presynaptic strength of the thalamic afferents onto LA PNs. To do this, we performed experiments where we stimulated the thalamic afferents in quick succession (20- and 100-ms interstimulus intervals) while recording EPSCs in the postsynaptic LA PN. These experiments revealed a selective increase in the paired-pulse ratio in LA PNs of *Fmr1*KO mice (two-way repeated measures ANOVA, *p*_Main Effect: genotype_ = 0.0465, *p*_Main Effect: interstimulus interval_ = 1.*56 × 10*^−5^, *n*_WT_ = 6, 4; *n_Fmr1_*_KO_ = 5, 3; Fig. 3*K*,*L*). Taken together, these data indicate a specific disruption of local E/I balance caused by a reduction in FFI onto local PNs in the *Fmr1*KO LA.

### Reduced FFI enhances synaptic plasticity during early development

**Figure 4.**
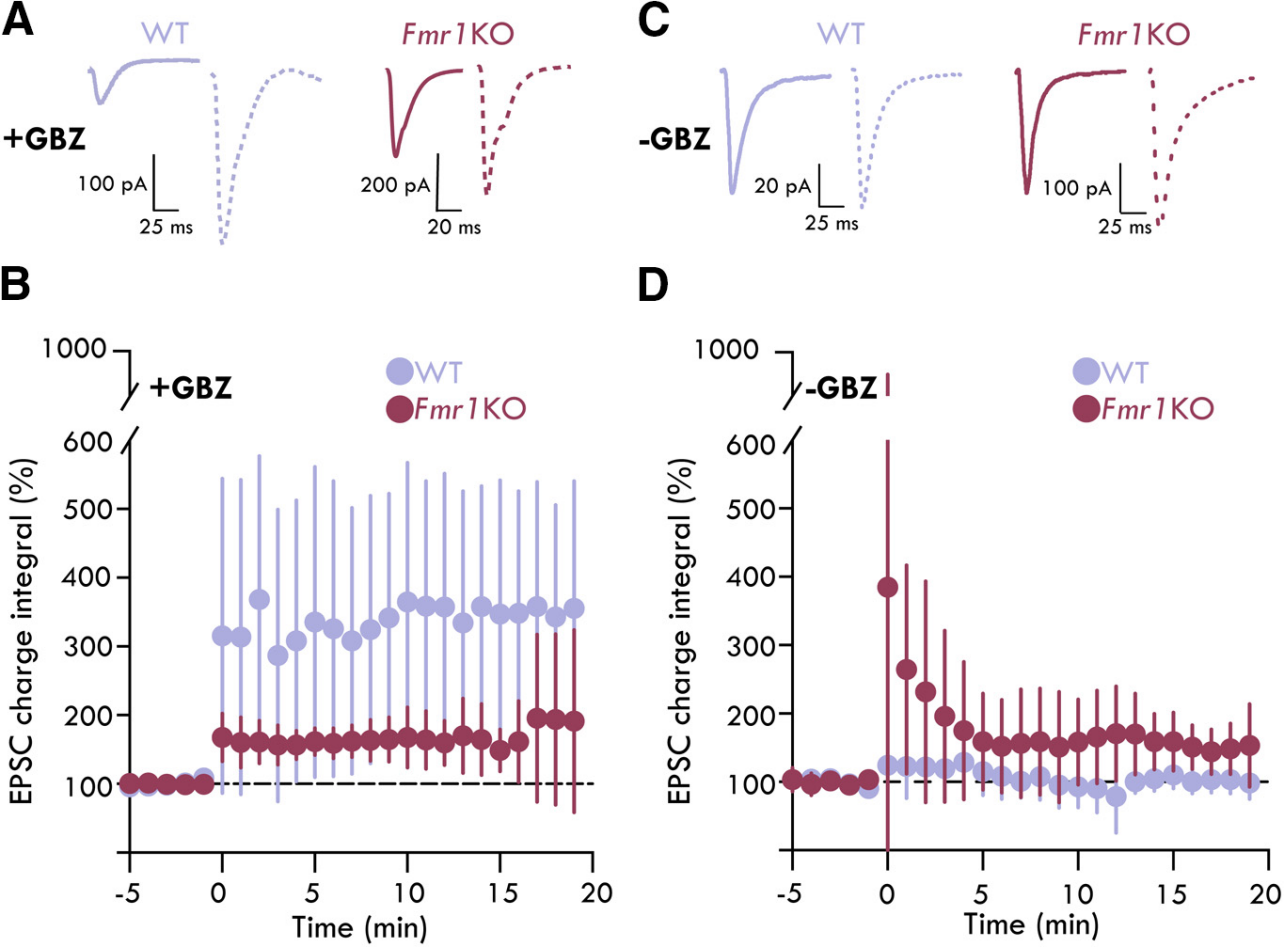
Aberrant LTP in *Fmr1*KO LA. ***A***, Representative mean EPSCs from LA PNs with 10 μm gbz. Solid lines show mean EPSCs before LTP induction and dashed lines show EPSCs at end of experiment. ***B***, Normalized EPSC charge integral across LTP experiments with 10 μms gbz. All PNs underwent significant LTP in both WT and *Fmr1*KO LA [unpaired *t* tests: *p*_WT1–6_ < 0.0001, *p_Fmr1_*_KO1_ < 0.0001, *p_Fmr1_*_KO2_ = 0.000200, *p_Fmr1_*_KO3_ = 0.000900, *p_Fmr1_*_KO4_ < 0.0001; *n*_WT_ = 6 neurons, 5 mice, 4 males (M), 1 female (F); *n_Fmr1_*_KO_ = 4, 4, 2 M, 2 F]. ***C***, Representative mean EPSCs from LA PNs without gbz. Solid lines show mean EPSCs before LTP induction and dashed lines show EPSCs at end of experiment. ***D***, Normalized EPSC charge integral across LTP experiments without gbz. One of six PNs underwent significant LTP, 2 PNs underwent LTD, four did not undergo significant LTP or LTD in WT LA. All PNs underwent LTP in *Fmr1*KO LA (unpaired *t* tests: *p*_WT1_ = 0.386, *p*_WT2_ = 0.142, *p*_WT3_ = 0.989, *p*_WT4_ = 0.0019, *p*_WT5_ = 0.0123, *p*_WT6_ < 0.0001. *p_Fmr1_*_KO1_ = 0.00540, *p_Fmr1_*_KO2–5_ < 0.0001; *n*_WT_ = 6, 4, 3 M, 1 F; *n_Fmr1_*_KO_ = 5, 3, 2 M, 1 F). Summary statistics presented as mean ± SD.

Local INs provide FFI onto PNs to gate LTP in BLA microcircuits (Bissière et al., 2003; Tully et al., 2007; Wolff et al., 2014; Bazelot et al., 2015), and LTP cannot be induced in PNs if local inhibition is intact (Bissière et al., 2003). In light of the observed reduction in FFI onto LA PNs in *Fmr1*KO mice (Fig. 3), we hypothesized that it would be possible to induce LTP in *Fmr1*KO LA without manipulating GABAergic neurotransmission. To test this hypothesis, we recorded EPSCs in LA PNs following stimulation of the internal capsule in the presence and absence of the GABA_A_ receptor blocker gabazine (gbz; 10 μm; Fig. 4). After a stable 5-min baseline recording (stimulation frequency = 0.066 Hz), a high-frequency tetanus stimulation was given to the internal capsule to induce LTP (2 trains of 100 pulses delivered at 100 Hz, 20 s apart). As expected, we observed LTP in all P21 WT PNs with gbz (unpaired *t* tests; Fig. 4*A*,*B*): EPSC charge integral [percent (%) change, MD ± SD], last 5 min_WTgrp_ = 501.30 ± 4.51%, *p*_WTgrp_ < 0.0001, MD: 401.30% CI: [390.90, 411.70], *n*_WT_ = 6 neurons, 5 mice, *n* = 2 FVB, 3 B6; EPSC charge integral_WT1_ = 468.43 ± 51.21%, *p*_WT1_ < 0.0001, MD: 368.43%, CI: [315.41, 421.71], *n*_WT1_ = 1, 1; EPSC charge integral_WT2_ = 508.84 ± 18.80%, *p*_WT2_ < 0.0001, MD: 408.84, CI: [387.00, 430.60]; *n*_WT2_ = 1, 1; EPSC charge integral_WT3_ = 276.52 ± 40.05%, *p*_WT3_ < 0.0001, MD: 176.52, CI:[132.30, 220.80]; *n*_WT3_ = 1, 1, EPSC charge integral_WT4_ = 140.30 ± 2.29%, *p*_WT4_ < 0.0001, MD: 40.30, CI:[36.13, 44.46]; *n*_WT4_ = 1, 1; EPSC charge integral_WT5_ = 1508.00 ± 19.16%, *p*_WT5_ < 0.0001, MD: 1408.00, CI:[1388.00, 1428.00]; *n*_WT5_ = 1, 1; EPSC charge integral_WT6_ = 105.80 ± 1.40%, *p*_WT6_ = 0.0003, MD: 5.80, CI:[3.55, 8.04]; *n*_WT6_ = 1, 1 (for how plasticity was determined, see Materials and Methods). Similarly, we observed LTP in all P21 *Fmr1*KO PNs with gbz (unpaired *t* tests; Fig. 4*A*,*B*): EPSC charge integral, last 5 min_Fmr1KOgrp_ 178.20 ± 9.69%, *p*_Fmr1KOgrp_ < 0.0001, MD: 78.20%, CI: [55.85, 100.57], *n_Fmr1_*_KO_ = 4, 4; *n* = 4 B6; EPSC charge integral_Fmr1KO1_ = 114.78 ± 3.82%, *p*_Fmr1KO1_ = 0.0001, MD: 14.78%, CI: [9.88, 19.66], *n_Fmr1_*_KO1_ = 1, 1; EPSC charge integral_Fmr1KO2_ = 122.25 ± 6.50%, *p*_Fmr1KO2_ = 0.0002, MD: 22.3%, CI: [14.58, 29.92], *n_Fmr1_*_KO2_ = 1, 1; EPSC charge integral_Fmr1KO3_ = 313.9 ± 93.6%, *p*_Fmr1KO3_ = 0.0009, MD: 213.9%, CI: [117.30, 310.50], *n_Fmr1_*_KO3_ = 1, 1; EPSC charge integral_Fmr1KO4_ = 161.90 ± 2.95%, *p*_Fmr1KO4_ < 0.0001, MD: 61.90%, CI: [51.38, 72.43], *n_Fmr1_*_KO4_ = 1, 1. However, in accordance with previous studies (Paradee et al., 1999; Zhao et al., 2005; Suvrathan et al., 2010), we observed a significant reduction in the magnitude of LTP in *Fmr1*KO PNs compared with WT LA PNs (unpaired *t* tests: EPSC charge integral, last 5 min_Fmr1KOvsWT_, *p*_Fmr1KOvsWT_ < 0.0001, MD: 172.0, CI: [148.0, 196.0], *n*_WT_ = 6 neurons, 5 mice, *n_Fmr1_*_KO_ = 4, 4).

Under conditions in which local inhibition remained intact, we found that LA PNs as a group did not undergo LTP in slices from WT mice (unpaired *t* tests; Fig. 4*C*,*D*): EPSC charge integral, last 5 min_WTgrp_ = 99.15 ± 3.25%, *p*_WTgrp_ = 0.80, MD: −0.84% CI: [−8.35, 6.65], *n*_WTgrp_ = 6, 5, *n* = 2 FVB, *n* = 3 B6, EPSC charge integral_WT1_ = 94.52 ± 5.07%, *p*_WT1_ = 0.39, MD: −5.48% CI: [−19.26, 8.30], *n*_WT1_ = 1, 1; EPSC charge integral_WT2_ = 114.04 ± 8.81%, MD: 14.04% CI: [−5.81, 33.89], *p*_WT2_ = 0.14, *n*_WT2_ = 1, 1; EPSC charge integral_WT3_ = 100.15 ± 16.84%, MD: 0.15% CI: [−22.99, 23.28], *p*_WT3_ = 0.99, *n*_WT3_ = 1, 1; EPSC charge integral_WT4_ = 119.84 ± 3.08%, *p*_WT4_ = 0.0019, MD: 19.84% CI: [9.72, 29.95], *n*_WT4_ = 1, 1; EPSC charge integral_WT5_ = 95.72 ± 2.22%, MD: −4.27% CI: [−7.34, −1.21], *p*_WT5_ = 0.0123, *n*_WT5_ = 1, 1; EPSC charge integral_WT6_ = 70.65 ± 1.12%, MD: −29.35% CI: [−35.79, −22.92], *p*_WT6_ < 0.0001, *n*_WT6_ = 1, 1. Interestingly, when we performed the same experiments in slices from *Fmr1*KO mice, we found that all neurons underwent significant LTP following high-frequency stimulation of thalamic afferents (unpaired *t* tests; Fig. 4*C*,*D*): EPSC charge integral, last 5 min_Fmr1KOgrp_ = 151.30 ± 2.96%, *p*_Fmr1KOgrp_ < 0.0001, MD: 51.30%, CI: [44.45, 58.11], *n_Fmr1_*_KOgrp_ = 5, 3; *n* = 1 FVB, *n* = 2 B6; EPSC charge integral_Fmr1KO1_ = 139.41 ± 13.0%, *p*_Fmr1KO1_ = 0.00540, MD: 39.41%, CI:[15.36, 63.46], *n_Fmr1_*_KO1_ = 1, 1; EPSC charge integral_Fmr1KO2_ = 223.90 ± 24.59%, *p*_Fmr1KO2_ = < 0.0001, MD: 123.90%, CI:[94.20, 153.60], *n_Fmr1_*_KO2_ = 1, 1; EPSC charge integral_Fmr1KO3_ = 126.55 ± 2.06%, *p*_Fmr1KO3_ = 0.0022, MD: 26.55%, CI:[12.76, 40.33], *n_Fmr1_*_KO3_ = 1, 1; EPSC charge integral_Fmr1KO4_ = 129.70 ± 3.34%, *p*_Fmr1KO4_ < 0.0001, MD: 29.70%, CI:[24.40, 34.96], *n_Fmr1_*_KO4_ = 1, 1; EPSC charge integral_Fmr1KO5_ = 136.90 ± 8.23%, *p*_Fmr1KO5_ < 0.0001, MD: 36.86%, CI: [27.49, 46.23], *n_Fmr1_*_KO5_ = 1, 1. Intriguingly, in +gbz condition, the magnitude of LTP in LA PNs in the *Fmr1*KO mouse exhibited only a modest increase (unpaired *t* tests; Fig. 4*B*,*D*): EPSC charge integral, last 5 min_Fmr1KO_GbzvsFmr1KO_noGbz_, *p*_Fmr1KO_GbzvsFmr1KO_noGbz_ = 0.027, *n_Fmr1_*_KO_Gbz_ = 4, 4, *n_Fmr1_*_KO_noGbz_ = 5, 3. Thus, the reduced FFI onto LA PNs in *Fmr1*KO mice correlated with a lower threshold of synaptic plasticity induction and a reduced magnitude of LTP in an important circuit for classical sensory-threat conditioning.

## Discussion

Synaptic dysfunction is a core aspect of NDDs (Chao et al., 2010; Zoghbi and Bear, 2012). In the present study, we investigated circuit function in a brain region known for reduced inhibitory neurotransmission in the *Fmr1*KO mouse model of FXS. Here, we show that PNs within the LA of *Fmr1*KO mice exhibit intrinsic membrane hyperexcitability over the P21–P35 developmental time point. Further, we show alterations in spontaneous and sensory afferent evoked excitatory and inhibitory neurotransmission. In particular, we demonstrate a preferential reduction in FFI onto PNs in the *Fmr1*KO LA. Finally, we find that the loss of FFI onto PNs and the increase in their excitability correlate with an enhancement of LTP at sensory afferents to the LA of *Fmr1*KO mice. Thus, we show coordinated changes in physiology, circuit function, and synaptic plasticity in a neural circuit responsible for sensory-threat learning.

### Increased excitability in LA may contribute to adverse behavioral symptoms in FXS

Within the LA, activity-dependent excitation of PNs underlies associative threat learning (Repa et al., 2001; Rosenkranz and Grace, 2002; Duvarci and Pare, 2014; Wolff et al., 2014). For instance, onset of the conditioned stimulus (CS) during classical Pavlovian threat conditioning elicits strong excitation of projection PNs. Increases in CS-evoked spike activity are observed in LA PNs after training (Quirk et al., 1995; Schoenbaum et al., 1999). Further, threat conditioning results in enhanced excitatory synaptic transmission of the auditory thalamic afferents onto PNs of the LA (McKernan and Shinnick-Gallagher, 1997; Namburi et al., 2015), and input-specific, Hebbian-like LTP underlies this synaptic strengthening (Nabavi et al., 2014; Kim and Cho, 2017).

Hyperexcitable PNs and the LA have been postulated to underpin a number of the neurologic and psychiatric symptoms in FXS and ASDs, as well as other neuropsychiatric disorders, including posttraumatic stress and anxiety disorders, attention-deficit/hyperactivity disorder, and substance use disorders (Posner et al., 2011; Contractor et al., 2015; Sharp, 2017). However, to date, few studies have focused on the amygdala of FXS patients in early life. Here, we demonstrate that PNs in the *Fmr1*KO LA exhibit marked hyperexcitability compared with WT. Specifically, LA PNs show increased maximum firing rates, a lower rheobase, and a more depolarized V_rest_. Thus, LA PN hyperexcitability may contribute to the clinical symptomatology of FXS. Previous studies in the hippocampus and across cortex identified similar hyperexcitable phenotypes in excitatory neurons of *Fmr1*KO mice resulting from alterations in HCN and voltage-gated Na^+^ and K^+^ ion channels (Gu et al., 2007; Higgs and Spain, 2011; Gonçalves et al., 2013; Zhang et al., 2014; Kalmbach et al., 2015; Deng and Klyachko, 2016; Routh et al., 2017). To our knowledge, similar detailed ion channel studies have not been performed in the LA of *Fmr1*KO mice. Thus, whether these channelopathies are region-specific or are also present in the LA of *Fmr1*KO mice remains to be determined. Future studies will be needed to completely define the ionic mechanisms underlying intrinsic excitability in PNs in the LA (Pape and Pare, 2010; Duvarci and Pare, 2014). Importantly, these studies may reveal new therapeutic targets for the treatment of anxiety disorders in FXS, ASDs, or other neuropsychiatric disorders.

### Alterations in inhibitory and excitatory synaptic strength underpin E/I imbalance

Globally, synaptic strength is modulated to maintain balanced excitatory and inhibitory activity within a network (Turrigiano, 1999). This synaptic scaling functions to maintain synaptic input in an activity-dependent manner (Turrigiano et al., 1998; Kilman et al., 2002). Our previous work has identified reductions in inhibitory synaptic efficiency and significant depletions in inhibitory function in PNs of *Fmr1*KO mice (Olmos-Serrano et al., 2010; Vislay et al., 2013; Martin et al., 2014) during P21–P30. Here, we extend these findings to include an enhancement of sEPSC amplitude in PNs in *Fmr1*KO LA. Taken together, these data suggest a circuit phenotype of enhanced excitability.

Additionally, we identified alterations in sIPSC decay kinetics and an enhancement of the frequency of sIPSCs onto local PNs. Previous work from our group identified alterations in GAT1-mediated GABA reuptake as well as GABA_A_R subunit-selective pharmacology demonstrating that GABA_A_R-dependent and independent mechanisms underlie changes in sIPSC kinetics at this time point (Vislay et al., 2013). Further, changes in sIPSC frequency and amplitude have been shown to result from AP-dependent increases in network activity (Vislay et al., 2013). While the precise receptor (or non-receptor) mechanisms underpinning the excitatory synaptic changes observed in our study remain to be determined, given the hyperexcitable phenotype of LA PNs in the *Fmr1*KO mouse it stands to reason that sEPSC amplitudes may be altered in an AP-dependent manner. Similarly, changes in sEPSC decay kinetics implicate receptor subunit compositions any of which may be altered in *Fmr1*KO mice (Li et al., 2002; Guo et al., 2015; Cheng et al., 2017). However, we cannot rule out changes in cell geometry or distribution of synapses. Future anatomic and biophysical studies will be needed to address potential sources of synaptic dysfunction. Interestingly, we did not observe similar changes in the decay kinetics of evoked responses. This may be because of the inherent mechanistic differences that underlie evoked versus spontaneous events. Primarily, evoked responses release increased concentrations of neurotransmitter at the synaptic cleft, engage different mechanisms of diffusion and re-uptake, and potentially engage extra-synaptic receptors (Thompson and Gähwiler, 1992; Draguhn and Heinemann, 1996). Further, alterations in synaptic currents may result from differences in synaptic structure, postsynaptic receptor composition and intrinsic conductances. As many of these mechanisms are affected in FXS (D’Hulst et al., 2009; Adusei et al., 2010; Olmos-Serrano et al., 2010), it is likely that changes in synaptic decay kinetics are masked in an evoked response as it is difficult to view a particular mechanism in isolation.

Unlike our previous studies with full excitatory synapse blockade (Olmos-Serrano et al., 2010; Vislay et al., 2013; Martin et al., 2014), in these studies, we used recording conditions that would enable the evaluation of both excitatory and inhibitory synaptic neurotransmission within the same cell. To do this, we filled pipettes with a CsMe intracellular solution which reduces resting and leak conductances and improves space-clamp. While imperfect, this method is superior to potassium-based internals for the measurement of more distal dendritic synapses that would normally be filtered (Williams and Mitchell, 2008). Thus, our synaptic recordings may have enabled better voltage control at more distal synapses, allowing us to evaluate additional sites of excitation and inhibition.

We speculate two possibilities for the role of enhancement of spontaneous, presynaptic inhibitory activity. First, it may function to compensate broadly for postsynaptic modulation of excitatory neurotransmission in a multiplicative manner (Turrigiano and Nelson, 2004) to maintain circuit homeostasis. Consistent with this, the magnitude of sEPSC amplitudes was increased in *Fmr1*KO LA PNs. Thus, increased sIPSC frequency may represent a compensatory homeostatic mechanism underlying gain control in the LA of *Fmr1*KO mice.

Alternatively, enhancement of spontaneous, presynaptic inhibitory activity may represent a homeostatic response to depleted FFI. FFI gates plasticity in the BLA circuit underlying learning of the sensory-threat associations (Bissière et al., 2003; Tully et al., 2007; Wolff et al., 2014; Bazelot et al., 2015). Importantly, disruption of this plasticity is believed to underlie the major pathophysiology of mood disorders such as anxiety and stress disorders (Duvarci and Pare, 2014). Our data reveal that at P21, evoked FFI is reduced in *Fmr1*KOs while evoked excitation is largely unaffected. Our results are in accordance with a recent study evaluating E/I conductance in the somatosensory cortex in the *Fmr1*KO mouse. In this study, a similar reduction in FFI in the L4→L2/3 feedforward circuit was observed coupled with a weaker decrease in feedforward excitation. Computational modeling using a parallel conductance model suggested that the overall net effect of this increase in E/I ratio was to maintain circuit homeostasis (Antoine et al., 2019). Thus, the increase in spontaneous inhibitory neurotransmission may represent a homeostatic mechanism to compensate for a loss of FFI in the LA of the *Fmr1*KO mouse. However, other studies in the *Fmr1*KO somatosensory cortex have demonstrated similar synaptic alterations with concomitant reductions in experience-dependent plasticity (Bureau et al., 2008; Harlow et al., 2010). Future studies should address the idiosyncrasies of how loss of *Fmr1* affects distinct neural circuits across the brain and their corresponding behaviors. Converging evidence from our group and others implicates LA Sst^+^ INs in FFI gating of LTP (Smith et al., 2000; Bissière et al., 2003; Unal et al., 2014; Wolff et al., 2014; Ito et al., 2019; Guthman et al., 2020). However, it remains to be shown if specific alterations in Sst^+^ IN function underlie the facilitated LTP seen in *Frm1*KO LA. Future studies will be needed to address IN function in a cell-type-specific manner in the *Fmr1*KO LA.

### E/I imbalance drives aberrant plasticity in the in the *Fmr1*KO LA

In our previously published work, we revealed that excitatory PNs in the *Fmr1*KO LA display a tendency toward narrower integration windows (Martin et al., 2014) that may imply decreased capacity for accurate input integration (Pouille and Scanziani, 2001; Isaacson and Scanziani, 2011) and plasticity in a circuit that is crucial for regulating fear and anxiety (Ehrlich et al., 2009). Indeed, numerous studies examining the neural correlates of amygdala-based behaviors in human FXS patients and mouse models have demonstrated reductions in amygdala function. In adolescents and adults with FXS, imaging studies conducted during the presentation of fearful stimuli demonstrated attenuated amygdala activation (Hessl et al., 2007, 2011; Kim et al., 2014). In the mouse model of FXS, previous studies of PNs in the LA of *Fmr1*KO mice have identified impairments in LTP (extracellular field recordings) in PNs in the LA (Paradee et al., 1999; Zhao et al., 2005; Suvrathan et al., 2010), reductions in the surface-expression of AMPA receptors (Suvrathan et al., 2010), and impairments in metabotropic glutamate receptor (mGluR)-mediated LTP, a process which modulates LTP in the LA under normal circumstances (Rodrigues et al., 2002). These plasticity deficits also occur in the context of presynaptic and postsynaptic deficits including reductions of both the frequency and amplitude of miniature excitatory postsynaptic currents and weakened excitatory presynapses (Suvrathan et al., 2010). However, these previous studies were conducted in older animals and employed LTP induction protocols in the presence of GABA_A_ receptor blockers. Thus, we could not directly compare these data to how the fluctuations of excitation and inhibition in the *Fmr1*KO mouse affects synaptic plasticity.

Since few studies have focused on emotional processing systems and how loss of the FMRP may affect circuit function and plasticity earlier in life, we evaluated LTP with and without GABA_A_ receptor blockers in younger animals to directly assess the inhibitory gating of synaptic plasticity in the juvenile LA of the *Fmr1*KO mouse. In accordance with the above-mentioned studies, under conditions of complete inhibitory blockade, we observed a reduction in the magnitude of LTP obtained in *Fmr1*KO LA PNs in the mouse compared with WT PNs suggesting that deficient plasticity emerges early in postnatal development. Most surprisingly, contrary to these reports of decreased synaptic plasticity in the LA of the *Fmr1*KO mice (Zhao et al., 2005), we observed that reduced FFI correlates with LTP in the thalamo-amygdalar circuit of juvenile *Fmr1*KO mice without inhibitory blockade. Thus, we postulate that lower threshold plasticity in the circuits responsible for fear-learning may underpin the pathophysiology of anxiety disorders in FXS and ASDs in early life. The mechanisms of fear extinction have been shown to be varied and complex (Myers and Davis, 2007). To the extent that fear extinction in the thalamo-amygdalar circuit is a process mediated by depotentiation (Kim et al., 2007; Hong et al., 2009), it is plausible that that loss of FMRP lowers the threshold for forming fear memories coupled with broad reductions in the efficacy of synaptic plasticity mechanisms underlying fear extinction. Thus, patients with FXS and ASD may be more prone to encode fear memories with a reduced ability to alter them. However, anxiety and fear-related disorders in FXS and ASDs may also be mediated by other mechanisms including changes in brain-wide functional connectivity (Haberl et al., 2015; Shen et al., 2016) or changes in neuromodulation (Hessl et al., 2002; Ghilan et al., 2015).

Of note, while both male and female animals were included in this study, male and female animals were not equally represented in our study populations which precluded rigorous sex difference analyses. Given the focus on sex as a biological variable, future studies examining sex differences are warranted. Regarding plasticity, future studies will be necessary to evaluate whether exogenously altering E/I balance, perhaps through the enhancement of inhibition, is capable of normalizing synaptic plasticity in the LA of the *Fmr1* KO mouse. Additionally, studies focused on plasticity mechanisms and behavioral studies related to fear retention and extinction are warranted. Further, changes in synaptic plasticity and fear-learning throughout early development will be needed to determine whether the trajectory of plastic changes seen in the juvenile BLA of *Fmr1* KO mice is pathologic or homeostatic.

## Synthesis

Reviewing Editor: Julie Bakker, University of Liege Decisions are customarily a result of the Reviewing Editor and the peer reviewers coming together and discussing their recommendations until a consensus is reached. When revisions are invited, a fact-based synthesis statement explaining their decision and outlining what is needed to prepare a revision will be listed below. The following reviewer(s) agreed to reveal their identity: NONE. Note: If this manuscript was transferred from JNeurosci and a decision was made to accept the manuscript without peer review, a brief statement to this effect will instead be what is listed below.

This ms has been transferred from the Journal of Neuroscience. The authors have replied to the previous comments made by 2 reviewers.

This has been done in a satisfactorily manner in general. However, some points remain. I strongly agree with “reviewer 1” that it would be important to identify neurons belonging to the same animal with either different symbols or shades in Figure 1 (but also for the other figures). This has not been changed by the authors, but I would strongly recommend doing so. The number of experimental animals remains rather low (3-4 for each genotype). So it’s important to see whether certain animals are actually outliers or not. Strictly statistically speaking, it’s not ok to use neurons coming from the same animal as individual data points.

Furthermore, the authors mentioned in the method section to have been using both male and female mice, but this is no longer discussed in the paper, nor are results presented separately for males and females. Were any differences observed between the two sexes. This should be discussed.

## Author Response

April 5, 2021

RE: Transfer of Manuscript: JN-RM-0274-21, by Svalina, Guthman et al., “Hyperexcitability and loss of

feedforward inhibition contribute to aberrant plasticity in the Fmr1KO amygdala.”

Dear Dr. Bakker:

We are grateful for the opportunity to publish our manuscript in eNeuro. Again, we would like to thank you, the

reviewing editors, and the peer reviewers for their imporant critiques. Unquestionably, this resulted in a stronger

and more rigorous manuscript. Further, we appreciate the opportunity to address the remaining concerns of the

editor and peer reviewers. Below we provide a point-by-point response that outlines how we addressed the these

concerns and outlines how corresponding results and edits have been incorporated into the revised manuscript.

Briefly, as suggested by both the editors and peer reviewers we have identified individual neuron data points as

belonging to specific animals, identified the number of males and females included in our studies, and remarked

on this in the discussion. We hope that our revisions make this manuscript suitable for publication in eNeuro.

Reviewers queries are in Italic font and responses immediately follow.

• “I strongly agree with “reviewer 1” that it would be important to identify neurons belonging to the same

animal with either different symbols or shades in Figure 1 (but also for the other figures). This has not been

changed by the authors, but I would strongly recommend doing so. The number of experimental animals

remains rather low (3-4 for each genotype). So it’s important to see whether certain animals are actually

outliers or not. Strictly statistically speaking, it’s not ok to use neurons coming from the same animal as

individual data points.”

Response: We have revised all figures to represent individual data points on a per animal basis. Data points

have been shaded, with each color represented a unique animal.

• “[T]he authors mentioned in the method section to have been using both male and female mice, but this is no

longer discussed in the paper, nor are results presented separately for males and females. Were any differences

observed between the two sexes. This should be discussed.”

Response: We have included the numbers of each sex included in our studies in the figure legends for all

figures. However, female and male animals were not equally represented in our study and this precludes our

ability to perform rigorous sex differences analyses. We have included mention of this limitation of our study in

the discussion [lines 1030 - 1033].Thank you very much for the opportunity to submit this revised manuscript.
